# The Principles and Practice of Dental Surgery

**Published:** 1850-12

**Authors:** 


### BIBLIOGRAPHICAL NOTICES.

---

Art. III.—*The Principles and Practice of Dental Surgery.* By Chapin A. Harris, M.D., D.D.S., Professor of the Principles and Practice of Dental Surgery in the Baltimore College; Fellow of the American Society of Dental Surgeons; Member of the American Medical Association, etc., etc. Fourth edition: revised, modified, and greatly enlarged. With two hundred Illustrations. Philadelphia: Lindsay & Blakiston. 1850.

We cheerfully welcome this valuable edition of Dr. Harris's work on Dental Surgery. The author has long been known as one of the most assiduous and capable cultivators of the science to which he is devoted, and, of course, has made himself one of the ablest. That a fourth edition of the professional work of such an author should have been called for so soon after the first, can excite no surprise among those who know the merits of the book, and the wants of that department of surgery, whose advancement it is intended to promote. But we earnestly hope that the study of this book will not be confined to the mere dental surgeon; there is scarcely a chapter of the work that is not replete with information essential to the general practitioner of medicine. His advice is often sought in dental affections, and he should be perfectly familiar with all their phenomena. He has, indeed, no more right to be ignorant of the large majority of the subjects, treated of in Dr. Harris's work, than he has to be ignorant of a knowledge of fractures, fever, etc. Those who rely on him for medical advice have as much right to look to him in Dental diseases as in any other affections. And we know of no work that is as well filled with useful information on dental subjects as the one before us.

The fourth edition has been considerably enlarged, and treats of some subjects that were omitted in former editions. These, however, belong more to the practical dentist, than the practitioner of medicine. One of these additions is on *filing teeth*, and we commend the judicious remarks of Dr. John Harris, on that subject, to the attention of the reader. The extremes on *filing teeth* must be avoided; it is a process that should not lightly be ventured on, yet, it is an operation that is often necessary and beneficial. There is no doubt that a sound, healthy enamel is a protection to a tooth, while it is quite as true that a diseased enamel can do no good, and may do much harm.

The author makes the following remarks, in the preface to his fourth edition: "The author indulges the hope that his efforts to keep pace with the progress of the art will be found satisfactory to his readers, and that independently of the advantages they may find from the added matter, they will be gratified to perceive in the necessity for it, the evidence of a progress in dentistry, most cheering to all who desire to see this branch of surgery rescued from the domain of ignorant empiricism."

"The great improvement made within the few years past gives good reason to expect that this department of medicine will soon receive the public and professional consideration it deserves."

Most cordially do we sympathise in all noble and elevated aspirations. The united labors of scientific dentists have already done much for the science and for the public, and all who love the profession must continue to labor for its advancement. Those who have watched its progress for the past twenty years, know how much good has been achieved; those who know its present condition know how much remains to be done. The profession of dental surgery is beset with swarms of charlatans of the lowest description, just as the profession of medicine is. An extensive system of "touting" prevails in both professions; the very countenances of these impudent, dishonest, swindling wretches, seem to proclaim the principle of the "patriot" John Wilkes—"the public is a goose, and he is a fool who will not pluck a feather." It is hoping too much, when we hope to see these outrageous things abolished, but the labors of the good, the wise, the honest and the benevolent may do much in mitigation of the evil. Those who love the profession of dental surgery or of medicine must advance it in their own lives; they must observe closely, reason truthfully, and study constantly to arm themselves with knowledge in every department of their science. It is not enough that a man has attained some initial honor, to which he looks as the basis of his respectability. He must do a great deal to show that he deserves the honor he has attained, and then a great deal more to rise to higher con ditions. The public have not that small modicum of discrimination for which the charlatan gives them credit, nor that amount which the qualified practitioner ascribes to tnem. They very soon detect the difference between qualifications and the want of them.

The labors of Dr. Harris and his companions have done a great deal for the advancement of dental surgery, and they deserve much credit for their labors. They have aimed at noble ends, and have achieved a large result. We wish them abundant prosperity in their future efforts.

This work is beautifully printed, and remarkably well illustrated. The publishers deserve much credit for their share of labor in this fourth edition. They have displayed an excellent taste and judgment in the manner in which they have discharged their duties, and men who so well deserve it, should have an abundant success in their business.

Among the illustrations, we are glad to see a drawing of the complete machine, invented by our fellow-citizen, Dr. Somerby, in which the powers of the blow-pipe and furnace are admirably combined. Dr. Harris says that Dr. Somerby has rendered a great service to the dental profession by this invention, and from what we have seen it do, we are at a loss to know how the particular mechanism in dentistry, to which it is devoted, ever managed to succeed in any degree without its services. Dr. Somerby deserves well for this successful application of mechanical ingenuity to the advancement of the profession, of which he is a distinguished ornament.

